# Supplementary material for: Clinical benefit of remdesivir in rhesus macaques infected with SARS-CoV-2
Source: bioRxiv. 2020 Apr 22:2020.04.15.043166. Preprint. [Version 2] doi: 10.1101/2020.04.15.043166 (PMC7239049; doi:10.1101/2020.04.15.043166)
Supplement: Supplement 1 [file media-1.pdf]

Supplementary materials

Clinical benefit of remdesivir in rhesus macaques infected with SARS-CoV-2

Brandi N. Williamson[1], MPH; Friederike Feldmann[2], AS; Benjamin Schwarz[3], PhD; Kimberly Meade-White[1], MSc; Danielle P. Porter[5], PhD; Jonathan Schulz[1], BSc; Neeltje van Doremalen[1], PhD; Ian Leighton, BA[3]; Claude Kwe Yinda[1], PhD; Lizzette Pérez-Pérez[1], MSc; Atsushi Okumura[1], DVM; Jamie Lovaglio[2], DVM; Patrick W. Hanley[2], DVM; Greg Saturday[2], DVM; Catharine M. Bosio[3], PhD; Sarah Anzick[4], PhD; Kent Barbian[4], MSc; Tomas Cihlar[5], PhD; Craig Martens[4], PhD; Dana P. Scott[2], DVM; Vincent J. Munster[1], PhD; Emmie de Wit[1*], PhD

[1]Laboratory of Virology, [2]Rocky Mountain Veterinary Branch, [3]Laboratory of Bacteriology and [4]Research Technologies Branch, National Institute of Allergy and Infectious Diseases, National Institutes of Health, Hamilton, MT, United States of America; [5]Gilead Sciences, Foster City, CA, United States of America

Table S1. Clinical and pathological observations in rhesus macaques inoculated with SARS-CoV-2 and treated with remdesivir.

Treatment	Animal	Clinical observations	Observations at necropsy
**Remdesivir**	RM1	Slightly decreased appetite	Mediastinal lymph nodes enlarged
	RM2	Slightly decreased appetite	None
	RM3	Slightly decreased appetite, pale appearance	Mediastinal lymph nodes enlarged
	RM4	Slightly decreased appetite, slightly dehydrated	Mediastinal lymph nodes enlarged
	RM5	Slightly decreased appetite	Mediastinal lymph nodes enlarged
	RM6	Mild dyspnea, pale appearance	Gross lung lesions; mediastinal lymph nodes enlarged
**Vehicle solution**	RM7	Piloerection, hunched posture, tachypnea, dyspnea, decreased appetite	Gross lung lesions; mediastinal lymph nodes enlarged; focal hemorrhage in colon
	RM8	Piloerection, hunched posture, tachypnea, dyspnea, decreased appetite	Gross lung lesions; mediastinal lymph nodes enlarged
	RM9	Piloerection, hunched posture, tachypnea, dyspnea, decreased appetite	Gross lung lesions; mediastinal lymph nodes enlarged
	RM10	Tachypnea, dyspnea, pale appearance, slightly dehydrated	Gross lung lesions; mediastinal lymph nodes enlarged
	RM11	Piloerection, tachypnea, dyspnea, decreased appetite, pale appearance	Gross lung lesions; mediastinal lymph nodes enlarged
	RM12	Piloerection, tachypnea, dyspnea, decreased appetite	Gross lung lesions; mediastinal lymph nodes enlarged; ~5ml fluid in peritoneum

Table S2. Deep sequencing results to confirm absence of known resistance mutations to remdesivir.

The timepoints for sequencing of BAL and swab samples were selected based on positivity in qRT-PCR to reflect the latest possible timepoints where the majority of animals were positive for that given sample type in qRT-PCR.

Treatment	Animal no.	Sample	Mean sequencing coverage	F476 (nt 14,878-14,880)	V553 (nt 15,109-15,111)
Remdesivir	RM1	BAL[a]	197.27	no variants	no variants
		LLLL[b]	21.05	no variants	no variants
		Nose swab[c]	138.89	no variants	no variants
		RLLL[d]	11.22	no variants	no variants
	RM2	BAL	21.7	no variants	no variants
		LLLL	32.65	no variants	no variants
		Nose swab	187.29	no variants	no variants
		RLLL	19.37	no variants	no variants
	RM3	BAL	4.76	ND[f]	ND
		LLLL	5.43	ND	ND
		Nose swab	49.11	no variants	no variants
		RLLL	2.08	no variants	ND
		Rectal swab[e]	35.47	no variants	no variants
	RM4	BAL	421.43	no variants	no variants
		LLLL	81.65	no variants	no variants
		Nose swab	31.97	no variants	no variants
		RLLL	0.05	ND	ND
		Rectal swab	0.56	ND	ND
	RM5	BAL	25.37	no variants	no variants
		LLLL	0.02	ND	ND
		Nose swab	25.13	no variants	no variants
		RLLL	0.31	ND	ND
	RM6	BAL	17.1	no variants	no variants
		LLLL	5.39	no variants	ND
		Nose swab	25.27	ND	no variants
		RLLL	1.25	ND	no variants
		Rectal swab	0.42	ND	ND
Vehicle	RM7	BAL	352.51	no variants	no variants
		LLLL	123.92	no variants	no variants
		Nose swab	1.11	ND	no variants
		RLLL	229.79	no variants	no variants
		Rectal swab	17.72	no variants	no variants
	RM8	BAL	80.01	no variants	no variants
		LLLL	16.3	no variants	no variants
		Nose swab	0	ND	ND
		RLLL	4.28	ND	ND
		Rectal swab	1.39	ND	no variants

RM9	BAL	258.39	no variants	no variants
	LLLL	1.34	ND	no variants
	Nose swab	0.87	ND	ND
	RLLL	26.65	no variants	no variants
RM10	BAL	250.71	no variants	no variants
	LLLL	7.12	no variants	no variants
	Nose swab	0.2	ND	ND
	RLLL	1210.31	no variants	no variants
	Rectal swab	5.17	ND	no variants
RM11	BAL	880.97	no variants	no variants
	LLLL	56.38	no variants	no variants
	Nose swab	2.63	ND	ND
	RLLL	597.65	no variants	no variants
	Rectal swab	0.1	ND	ND
RM12	BAL	415.3	no variants	no variants
	LLLL	0.43	ND	ND
	Nose swab	20.44	no variants	no variants
	RLLL	88.08	no variants	no variants
	Rectal swab	11.56	ND	no variants

[a] BAL: bronchoalveolar lavages collected at 3 dpi.

[b] LLLL: left lower lung lobe collected on 7 dpi

[c] collected on 5 dpi

[d] RLLL: right lower lung lobe collected on 7 dpi

[e] collected on 2 dpi

[f] No sequence coverage or coverage was too limited to call

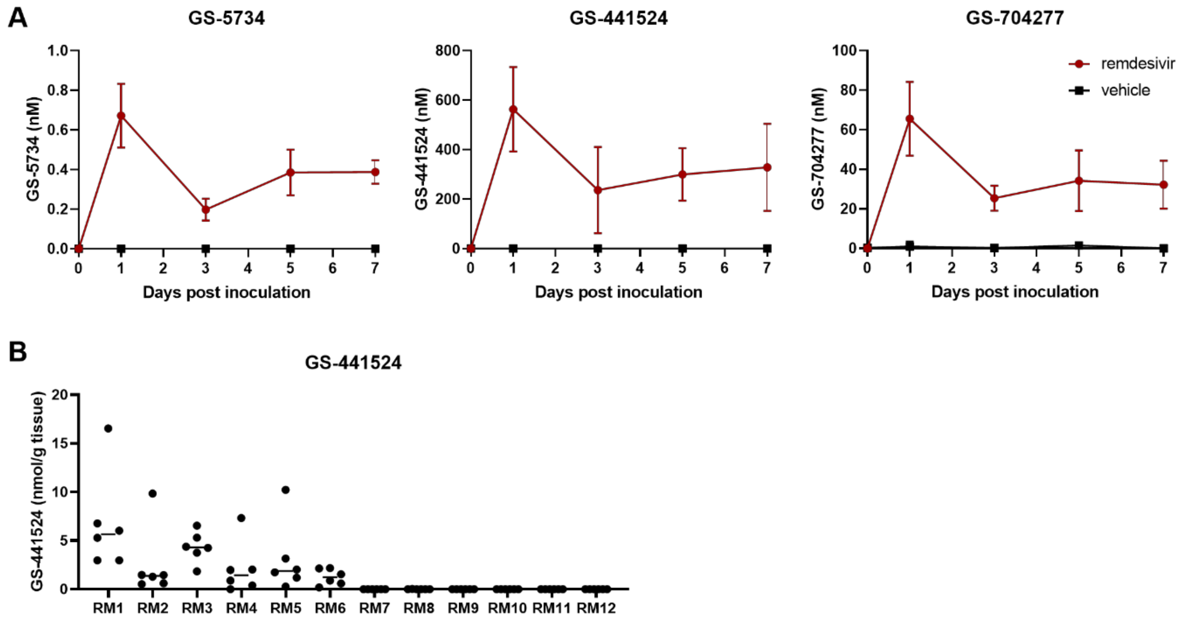

Figure S1. Concentration of remdesivir prodrug and metabolites measured in serum and lung homogenates of rhesus macaques infected with SARS-CoV-2. Two groups of six rhesus macaques were inoculated with SARS-CoV-2 strain nCoV-WA1-2020. Twelve hours post inoculation, one group was administered 10mg/kg intravenous remdesivir and the other group was treated with an equal volume of vehicle solution (2ml/kg). Treatment was continued 12hrs after the first treatment, and every 24 hrs thereafter with a dose of 5 mg/kg remdesivir or equal volume of vehicle solution (1ml/kg). Panel A shows the serum concentration of remdesivir prodrug GS-5734, the dephosphorylated nucleoside product GS-441524 and the intermediate alanine metabolite GS-704277 over time as measured in by LCMS. Mean and standard deviation are shown. Panel B shows the concentration of GS-441524 homogenized lung tissue collected from all six lung lobes on 7 dpi, 24 hrs after the last remdesivir treatment was administered. Each dot represents the concentration of GS-441524 in one lung lobe.
